# Iterative Large Language Model–Guided Sampling and Expert-Annotated Benchmark Corpus for Harmful Suicide Content Detection: Development and Validation Study

**DOI:** 10.2196/73725

**Published:** 2026-02-05

**Authors:** Kyumin Park, Myung Jae Baik, YeongJun Hwang, Yen Shin, HoJae Lee, Ruda Lee, Sang Min Lee, Je Young Hannah Sun, Ah Rah Lee, Si Yeun Yoon, Dong-ho Lee, Jihyung Moon, JinYeong Bak, Kyunghyun Cho, Jong-Woo Paik, Sungjoon Park

**Affiliations:** 1 SoftlyAI, Seoul, Republic of Korea; 2 Department of Psychiatry, Kyung Hee University College of Medicine, Seoul, Republic of Korea; 3 Department of Artificial Intelligence, Sungkyunkwan University, Office 27306, Engineering Building 2, 2066 Seobu-ro Jangan-gu, Suwon-si, Gyeonggi-do, 16419, Republic of Korea, +82 31 290 7104; 4 KAIST, Daejeon, Republic of Korea; 5 Department of Psychology, University of Pennsylvania, Philadelphia, PA, United States; 6 Department of Computer Science, New York University, New York, NY, United States

**Keywords:** artificial intelligence, dataset, suicide, suicide-related content, large language models

## Abstract

**Background:**

Harmful suicide content on the internet poses significant risks, as it can induce suicidal thoughts and behaviors, particularly among vulnerable populations. Despite global efforts, existing moderation approaches remain insufficient, especially in high-risk regions such as South Korea, which has the highest suicide rate among Organisation for Economic Co-operation and Development countries. Previous research has primarily focused on assessing the suicide risk of the authors who wrote the content rather than the harmfulness of content itself which potentially leads the readers to self-harm or suicide, highlighting a critical gap in current approaches. Our study addresses this gap by shifting the focus from assessing the suicide risk of content authors to evaluating the harmfulness of the content itself and its potential to induce suicide risk among readers.

**Objective:**

This study aimed to develop an artificial intelligence (AI)–driven system for classifying online suicide-related content into 5 levels: illegal, harmful, potentially harmful, harmless, and non–suicide-related. In addition, the researchers construct a multimodal benchmark dataset with expert annotations to improve content moderation and assist AI models in detecting and regulating harmful content more effectively.

**Methods:**

We collected 43,244 user-generated posts from various online sources, including social media, question and answer (Q&A) platforms, and online communities. To reduce the workload on human annotators, GPT-4 was used for preannotation, filtering, and categorizing content before manual review by medical professionals. A task description document ensured consistency in classification. Ultimately, a benchmark dataset of 452 manually labeled entries was developed, including both Korean and English versions, to support AI-based moderation. The study also evaluated zero-shot and few-shot learning to determine the best AI approach for detecting harmful content.

**Results:**

The multimodal benchmark dataset showed that GPT-4 achieved the highest *F*_1_-scores (66.46 for illegal and 77.09 for harmful content detection). Image descriptions improved classification accuracy, while directly using raw images slightly decreased performance. Few-shot learning significantly enhanced detection, demonstrating that small but high-quality datasets could improve AI-driven moderation. However, translation challenges were observed, particularly in suicide-related slang and abbreviations, which were sometimes inaccurately conveyed in the English benchmark.

**Conclusions:**

This study provides a high-quality benchmark for AI-based suicide content detection, proving that large language models can effectively assist in content moderation while reducing the burden on human moderators. Future work will focus on enhancing real-time detection and improving the handling of subtle or disguised harmful content.

## Introduction

*Harmful suicide content* on the internet poses a significant risk because it can induce suicidal thoughts in readers, potentially leading to self-harm or suicide [1,2]. The harmful suicide content includes materials that encourage or glorify suicide [3], making it appear as an attractive option and sharing suicide methods or instilling suicide knowledge in individuals with suicidal thoughts, thereby increasing the likelihood of actual suicide attempts [4]. In some cases, exposure to such harmful suicide content has led middle school students to commit suicide [5]. An analysis of adolescent suicide cases reveals that this age group, particularly female adolescents, is more vulnerable to the influence of triggering content [6,7]. Therefore, it is crucial to moderate such harmful suicide content before it spreads extensively.

Therefore, efforts to moderate harmful suicide content are intensifying. In the United States, initiatives focus on raising public awareness and safe content distribution, aligning with the World Health Organization guidelines [8]. Meanwhile, in 2022, the United Kingdom passed a law that makes such content illegal, emphasizing its serious commitment to addressing this issue [9]. In the Republic of Korea, which has the highest suicide rates among Organisation for Economic Co-operation and Development countries [10], the National Assembly of the Republic of Korea amended the Suicide Prevention Act, and the government has declared the dissemination of such content as illegal since 2019 [2]. Despite the increasing spread of harmful suicide content, its moderation is currently handled by only a single official and fewer than a thousand volunteers [11,12]. Considering the extensive use of social media in Korea [13], monitoring the large amounts of content is extremely challenging. In addition, moderating suicide content often leads to a high level of mental stress, hindering their ability to consistently and effectively monitor such content. Therefore, the need for an automatic harmful suicide content moderation system is urgent. The system can efficiently manage a growing volume of the content and ease the burden on human moderators.

However, previous research on online suicide content primarily focused on predicting the suicide risk of the authors who wrote the content. Zirikly et al [14] classified the suicide risk of authors based on content posted online (Reddit) into 4 levels. Similarly, Milne et al [15] and Yates et al [16] conducted research to predict the suicide and self-harm risks of online content authors. Subsequently, Yang et al [17] and Sawhney et al [18] used weakly supervised learning to enhance detection performance or collaborated with clinicians. Furthermore, Rawat et al [19] and Sawhney et al [20] performed tasks to detect suicide ideation and suicide events. All these studies focused on detecting the suicide risk of the author presented in the posts; they did not consider the potential propagation of suicide risk to readers by increasing their susceptibility to self-harm or suicidal behaviors. Other studies focused on understanding the negative effects of suicide content [6,21], or identifying the individuals who are most affected by the content [22-25]. Studies among Chinese adolescents have shown significant correlations between digital media usage and suicide or self-harm [23], and a meaningful relationship between suicide cases among Korean youths and searches related to suicide or self-harm [25]. In addition, three-quarters of young adults who have attempted suicide have reported using the internet for suicide or self-harm–related reasons [26], highlighting the risk posed by information that can induce suicide or self-harm.

Therefore, we introduce a *harmful suicide content detection* task that determines the level of harmfulness of the harmful suicide content to viewers. We then develop a multimodal *harmful suicide content detection benchmark* and a *task description document*. This document contains detailed instructions for annotators on how to assess the harmfulness of suicide-related content, which could also be useful for building instructions for large language models (LLMs). The benchmark and the document are developed by medical professionals, because such content might involve harmful visual language information that requires the judgment of the professionals (eg, self-harm photographs, or name of illegal drugs that can be used for suicide). Because labeling harmful suicide content causes mental stress, we focus on creating a small yet high-quality dataset. Furthermore, we demonstrate various methods using LLMs that can be effectively performed with few-shot examples.

Our contributions are as follows: (1) We propose a harmful suicide content detection task that classifies multimodal harmful suicide content as illegal, harmful, potentially harmful, harmless, or non–suicide-related. (2) We build a harmful suicide content detection benchmark that is multimodal and a Korean benchmark of 452 curated user-generated contents with corresponding medical expert annotations and a detailed task description document including the task details and instructions for annotators. (3) We create an English harmful suicide content detection benchmark translated from the Korean benchmark using a model (GPT-4) and analyze the quality and issues of translating suicide contents. (5) We demonstrate strategies to use LLMs to detect harmful suicide content by using the task description document and a small yet high-quality harmful suicide content detection benchmark. We test various closed and open-sourced LLMs using the machine-translated English benchmark. We observe that GPT-4 achieves *F*_1_-scores of 66.46 and 77.09 in detecting illegal and harmful suicide content, respectively.

## Methods

### Study Design

In this section, we describe the design process for the harmful suicide content detection task and the steps to construct a harmful suicide content detection benchmark for this task. First, in the “Task Design” subsection, we outline the process of designing the task with emphasis on real-world implementation for detecting harmful suicide content in real time from online sources. This design phase includes detailed considerations for building the dataset.

Next, the “Data Processing Workflow” subsection describes the end-to-end process, from collecting harmful suicide content to developing the final harmful suicide content detection benchmark. Harmful suicide content is gathered from various online sources, and the preannotation process [27,28], which uses LLMs, is used to reduce annotators’ exposure to vast quantities of harmful suicide content. Feedback generated from the annotation process by medical experts is used to update the task description document, which includes the categories and descriptions of harmful suicide content. This task description document is then used as an instruction for the preannotation process, creating an iterative process that builds and refines the harmful suicide content detection benchmark. Through this process, we ensure the inclusion of diverse, accurately labeled real-world harmful suicide content in the harmful suicide content detection benchmark while constructing a task description document that provides precise and comprehensive classification and explanations of the harmful suicide content.

As a result, from a collection of 43,244 harmful suicide content data points, we created a harmful suicide content detection benchmark consisting of 452 data points annotated by medical experts with suicide content categories, subcategories, and rationales for their classifications.

### Ethical Considerations

We obtained institutional review board (IRB) approval (approval number: KHUH 2023-04-072) from the IRB of Kyung-Hee University Hospital, South Korea, based on the principles of the Declaration of Helsinki, ensuring adherence to ethical standards. The IRB approval includes the collection of suicide-related data, the development of guidelines for data classification, and data labeling. It also specifies the acquisition of data through the collection of publicly available posts from online spaces targeting the general public, and it outlines the development of an artificial intelligence model for detecting suicide-related content using these data. All personally identifiable information (PII) (eg, user IDs, names, addresses, and phone numbers) was anonymized and replaced with standardized tags. The benchmark contains extremely disturbing text and images, including self-harming photographs, blood, tools used for suicide and self-harm, and drug information. Even among medical professionals and researchers, prolonged exposure to such images can lead to severe mental stress. Therefore, we have deliberately chosen not to aim for the creation of a large-scale dataset but rather to limit the workload to prevent further intensifying mental stress. All these processes were conducted with IRB approval obtained prior to data collection. Given the nature of harmful suicide content and the legal restrictions against its unrestricted distribution, it is challenging to share the benchmark dataset openly. We understand the legal implications of distributing data containing information that potentially induces suicide. Despite these concerns, we believe that collecting such data to build a benchmark and conducting research to prevent its spread on the internet outweigh these legal issues. Access to the benchmark will be strictly limited, allowing only those researchers with IRB approval and a commitment not to distribute the content further, ensuring responsible use for research purposes only and adherence to legal standards. We believe that our work contributes significantly to the ongoing international effort against harmful suicide content and hope to aid in preventing its spread on the internet.

### Task Design

**Figure 1.**
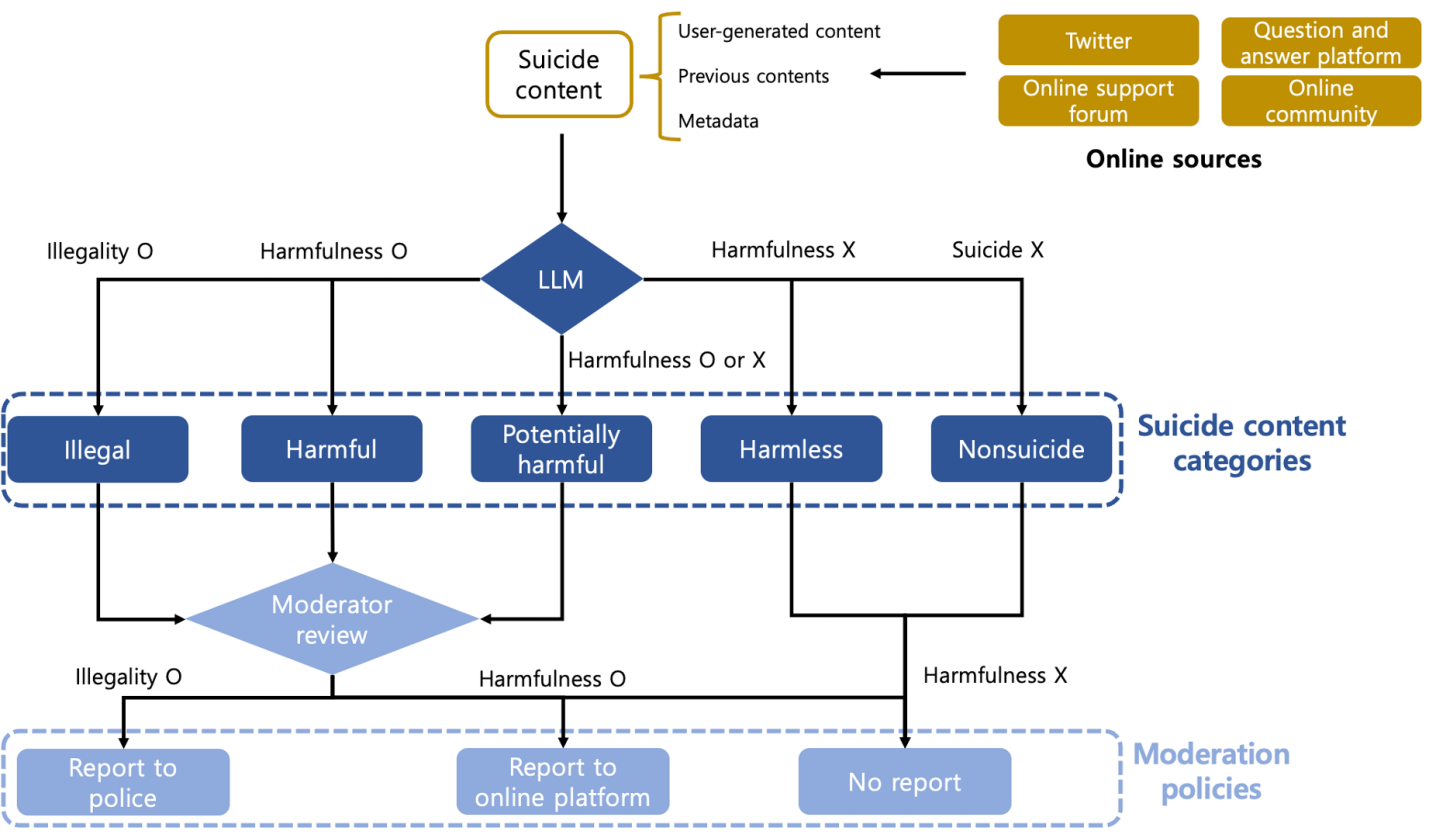
Moderation system for harmful suicide content detection, categorizing online user-generated content into 5 classes by legality, harmfulness, and suicide relation. A moderator reviews content with potential illegality or harm, leading to legal reporting or content removal requests. No action is taken if no risks are found. “O” indicates that the corresponding value exists, and “X” indicates that the value does not exist. “O/X” denotes the potentiality of whether the corresponding value exists or not. LLM: large language model.

Figure 1 illustrates the concept of using harmful suicide content detection in a real-world *moderation system*. The moderation system uses a model to automatically detect harmful suicide content and checks for illegal or harmful content and implements the appropriate *moderation policy* through a moderator’s review. As this study introduces the harmful suicide content detection for the first time, our focus is on developing a model to automatically detect harmful suicide content rather than implementing an end-to-end moderation system. Therefore, this study focused on developing a harmful suicide content detection with this moderation system in mind, leaving the implementation of an end-to-end moderation system for future work. Specifically, we considered the inputs and outputs of harmful suicide content detection, considering the various real-world information on harmful suicide content that a moderation system might encounter as well as the moderation actions by a moderator.

The model classifies the content into 5 distinct *suicide categories* based on illegality, harmfulness, and suicide-related aspects. For categories identified as having minimal harmfulness, a moderator validates the harmfulness through a review. Finally, the moderator moderates harmful suicide content using a moderation policy suitable for the identified harmfulness and illegality of the content, thereby minimizing human intervention and effectively moderating harmful suicide content.

To ensure that the detection system is applicable to real-world online data, the task targets various data from diverse sources to encompass a broad spectrum of user-generated content encountered on the internet. This approach ensures that the system effectively addresses the complexities and nuances of online posts (“Task Input” section).

The classification results produced by the harmful suicide content detection were crafted considering the functionality of a moderation system in mind. This means that the categories into which the content is sorted are specifically designed to facilitate the practical use of these results in moderating the content, ensuring that the system can serve as an effective tool for maintaining online safety and supporting mental well-being (“Task Output” section).

A moderator review was designed to validate the model’s classification results and implement appropriate moderation. Specifically, for content categorized under the harmful categories, the process validates the harmfulness to ensure the reliability of the moderation system. In addition, because this process involves confirming the results of the model rather than newly identifying and classifying suicide content, it is more efficient in terms of reducing mental stress on moderators. Finally, suitable moderation policies were implemented based on the validation results. This system was developed to regulate suicide content and execute different moderation policies based on the illegality and harmfulness, thus preventing the spread of harmful suicide content online (“Moderator Review” section).

In summary, the entire process involves the model classifying online content into suicide categories, the moderator reviewing the results, and then implementing the corresponding moderation policy to ensure that the moderation system functions effectively. Throughout this process, multimodality information such as text and image data are used to reflect various aspects of the content in the model’s input. Metadata from diverse sources and previous content serve as context. The model’s output comprises 5 suicide categories, each differentiated by the presence or absence of illegality, harmfulness, and suicide-related aspects. Finally, the moderator review efficiently uses the model’s classification results for validation, and effective moderation policies are implemented based on the content’s illegality and harmfulness, thereby preventing the spread of harmful suicide content online.

### Task Input

#### Considerations

We consider the following for designing the input of the task. (1) *Multimodality*: Because 50% of harmful suicide content contain images or videos [29], we consider text and images as inputs. (2) *Source diversity*: Harmful suicide content appears across various platforms, from social media to online communities [29]. We collected data from diverse sources for comprehensive coverage. (3) *Context information*: We also incorporate previous content and metadata into our inputs. Previous content reveals the context of the target content, whereas metadata, such as user descriptions and view counts, provides additional insights, aiding in accurate harmfulness assessment.

#### Inputs

Given the considerations, the inputs for the task are as follows: (1) *User-generated content*: Contents created by users. The content includes text, and possibly images and URLs. Images and URLs are converted to text manually or using machine learning models (eg, image captioning and summarization). (2) *Previous content*: Previous content is often required because it provides context, clarifies references, and provides background information essential for the full comprehension of user-generated content. (3) *Metadata*: Other contextual information about the user-generated content such as view counts, like counts, creation time, user self-description, and so on.

### Task Output

#### Considerations

We consider the following for designing the outputs of the task. (1) *Expert judgment*: Harmful suicide content involves specific terminology related to suicide, such as professional drug names, slang, and abbreviations. Thus, clinical expertise is required to accurately determine the legality and harmfulness of such suicide content and to decide on an appropriate response to the content. (2) *Moderation policy*: If an automatic harmful suicide content detection model is developed, it should be part of a moderation system and collaborate with human moderators or domain experts [18]. This implies that once the model detects harmful suicide content, it is necessary to consider appropriate actions. Therefore, the response of each output was considered when defining the output.

#### Outputs

We develop five harmful suicide content categories. Content should be mapped to one of the following categories: (1) *illegal* content that encourages or assists suicidal behavior; (2) *legal but harmful* content that, while not illegal, significantly induces suicide; (3) *potentially harmful* content that could be triggering for certain individuals, whereas it may be benign for others; (4) *harmless* content that is either neutral or positive for suicide; and (5) *nonsuicide* content that is not related to suicide.

### Moderator Review

Moderator review includes the process of reexamining the suicide content classified by the model using moderator (eg, a clinical expert) and implementing the appropriate moderation policy. The moderation system identifies the harmfulness and illegality of suicide content and implements a corresponding moderation policy to block the spread of such content online. Thus, through moderator review, the moderator (1) verifies the classification result of the model and (2) implements the corresponding moderation policy. The moderator review is conducted for results classified as illegal, harmful, and potentially harmful because most online information is unrelated to suicide and reviewing all the information would increase moderator fatigue. Hence, reviews are conducted only for suicide information that may cause harm. The moderator verifies the illegality and harmfulness of content within these categories and conducts the corresponding moderation policy. Therefore, the moderator requires knowledge to comprehend and understand the content and distinctions of suicide content.

#### Moderation Policies

The moderator reviews the model’s classification results and implements a corresponding moderation policy. The moderation policies are as follows: (1) *Report to police*: This is the strongest form of moderation policy intended to subject content creators to legal regulations by reporting to legal institutions. (2) *Report to online source*: Reporting the content to the online source where it is posted intends to prevent the spread of harmful information by requesting the deletion of the content. (3) *No report*: No additional actions, such as reporting the posts, are taken, allowing it to circulate online.

*Report to police* responds to content within the illegal suicide category containing illegal information. According to Korean law, certain types of content related to suicide are defined as illegal. Such content often includes illegal activities, such as the sale of illegal drugs; hence, reporting to legal institutions (eg, the police) imposes legal sanctions on the poster of such content.

*Report to online source* prevents the online spread of content containing or potentially containing harmful information related to suicide, such as illegal, harmful, and potentially harmful content. Because information spreads quickly online, it is reported to the online source where it was posted, and its removal is requested to prevent dissemination. For potentially harmful information, the harmfulness of which can vary depending on the reader, the moderator assesses the degree of harmfulness and reports whether it is severe.

*No report* is for harmless or nonsuicide content that poses no problem when posted online. Most online content is unrelated to suicide; therefore, it does not require reporting.

Table 1 shows the features of each category in terms of legality, harmfulness, and association with suicide. *Illegal suicide content* contains the most dangerous information, explicitly encouraging or facilitating suicidal behaviors. This category is critical for immediate intervention, embodying content that can actively propel individuals toward self-harm or suicide. *Harmful suicide content*, while not directly inciting suicide, significantly affects the audience by portraying suicide or self-harm in a manner that can trigger such actions among vulnerable individuals. The specificity of the depiction, whether through graphic imagery or detailed descriptions, amplifies its potential harm, making it a crucial target for moderating content. *Potentially harmful suicide content* traverses a gray area, with content that might not universally trigger harmful behaviors but could potentially do so in susceptible populations. This category underscores the complex challenge of content moderation, in which the impact of the content is not universally harmful and may vary significantly among individuals. *Harmless suicide content* focuses on providing support, hope, or neutral information regarding suicide without posing a risk of harm. This category plays an essential role in suicide prevention by offering resources, support, and information to reduce suicide rates. Finally, *nonsuicide content* serves as a catchall for material that does not pertain to suicide or self-harm, highlighting the importance of distinguishing between genuinely harmful content and content unrelated to suicide.

**Table 1.** Illegality, harmfulness, and suicide relativity of categories and the moderation protocols^a^.

Category	Illegality	Harmfulness	Suicide- related	Moderator review	Moderation policy
Illegal	O	O	O	O	Report to police and online source
Harmful	X	O	O	O	Report to online source
Potentially harmful	X	Δ	O	O	Report to online source or no report
Harmless	X	X	O	X	No report
Nonsuicide	X	N/A^b^	X	X	No report

a The (Δ) symbol represents a state that is in a gray area, indicating that the characteristic is neither fully present nor completely absent. "O" denotes the presence or applicability of a given attribute, whereas "X" denotes its absence. For instance, data categorized as "Harmful" are characterized by the absence of illegality (X), the presence of harmfulness (O), suicide-related (O), and the moderator review (O).

b N/A: not applicable.

### Data Processing Workflow

**Figure 2.**
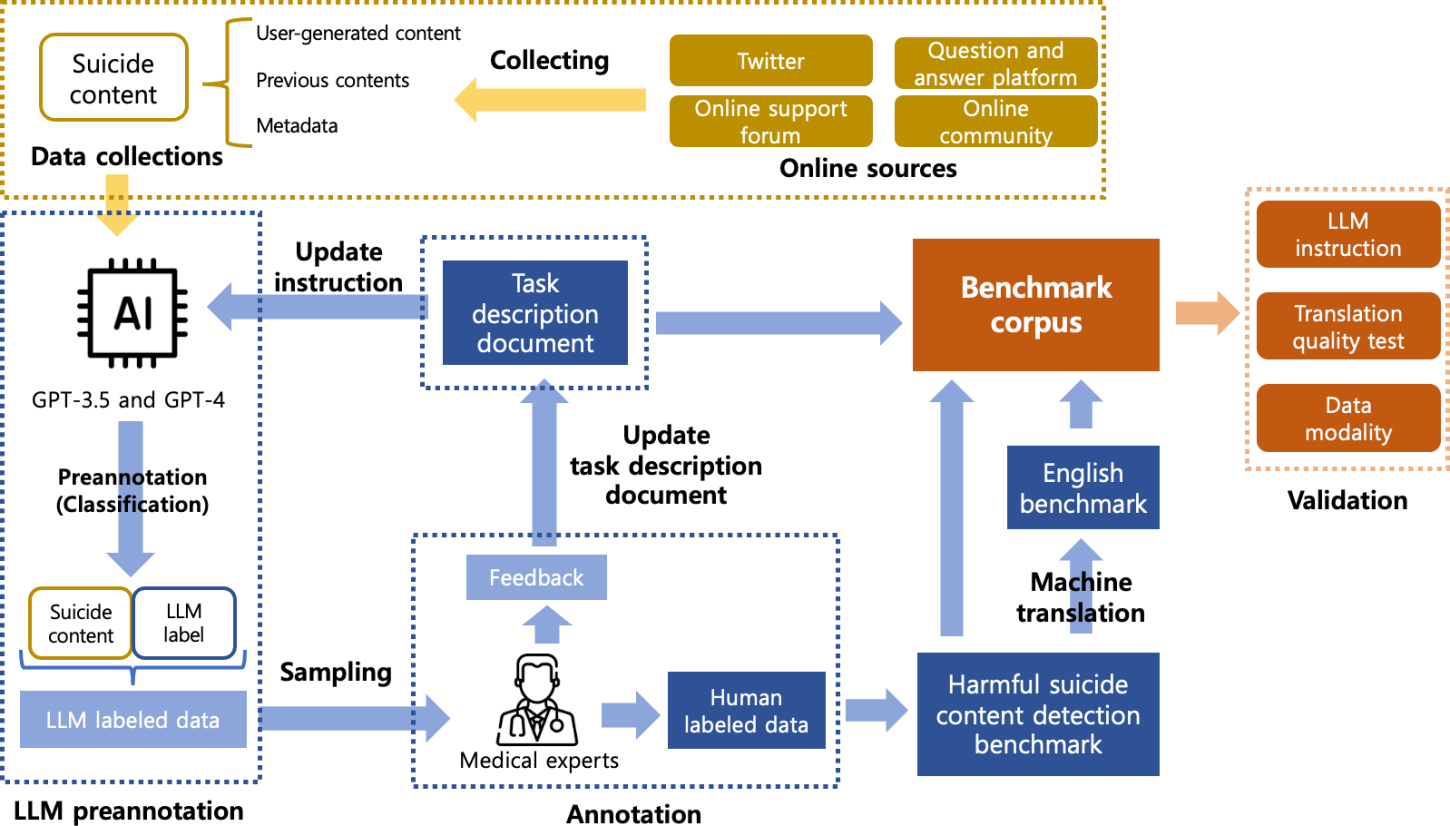
Overview of the data processing workflow, which consists of data collection, benchmark construction, and benchmark validation. We collect suicide content and annotate it with medical experts, leveraging LLM-based preannotation to balance categories, and we iteratively refine the task description document through feedback. We then construct the benchmark corpus with human-labeled data, an English-translated version, and the updated task description, and we validate it for applicability to LLMs. AI: artificial intelligence; LLM: large language model.

This section describes the data processing workflow used to create a high-quality harmful suicide content detection benchmark for detecting harmful suicide content. As illustrated in Figure 2, the workflow starts from data collection, detailed in “Data Collections” subsection, and outlines the process to develop a robust and diverse benchmark for harmful suicide content detection, described in “Data Annotation” subsection. The primary goal is to create a small but high-quality benchmark that can comprehensively capture real-world scenarios and task description document that can be effectively used for LLM instruction. Key challenges include the stressful nature of the annotation process, the extremely low prevalence of illegal suicide content, and the need to ensure the benchmark’s completeness and use, given that this is a newly proposed task that must reflect various aspects of real-world data.

The methods used to address these challenges include (1) LLM sampling to reduce annotator exposure to a large volume of harmful suicide content while increasing the representation of rare, illegal suicide content; (2) an iterative annotation approach to continuously update the task description document and incorporate real-world scenarios, aiming to reflect diverse data until no further updates are required, while also considering modality (image and context) to adapt annotators effectively; and (3) filtering and validation, which involve updating the harmful suicide content detection benchmark to align with the latest task description document, categorizing and subcategorizing data accurately, and removing outliers such as incomprehensible content.

#### Data Collections

Developing a large-scale harmful suicide content dataset is highly challenging. Harmful suicide content is infrequently encountered in real-world scenarios [30], and the distressing nature of such content can cause mental strain for annotators. In addition, obtaining annotations from medical experts is expensive. Therefore, we focus on developing a high-quality curated benchmark dataset. Prior to the dataset collection, we obtained approval from the IRB of Kyung-Hee University Hospital, South Korea.

To cover the diverse source domains of the content, we collect user-generated content related to suicide from social media, Q&A platforms, online support forums, and online communities. Table 2 lists the number of raw data, benchmark data, descriptions of content, previous content, and metadata of each source.

**Table 2.** Number of collected harmful suicide content and harmful suicide content detection benchmark dataset for each domain^a^.

Source	Raw data (no. of images)	Benchmark (no. of images)	Content	Previous content	Metadata
Twitter	12,125 (3,671)	359 (78)	Tweets written by users	Previous tweets in the thread where the content is written	User description, view count, like count, etc
Online community	794 (794)	48 (48)	Title and bodies of post written by users	N/A^b^	User nickname, view count
Q&A^c^ platform	13,104 (0)	33 (0)	Question or answers written by users	Questions (if the content is an answer)	N/A
Online support forum	17,325 (0)	23 (0)	Counseling request posts or responses written by users or counselors	Counseling request posts (if the content is a response)	N/A
Total	43,244 (4,429)	452 (126)	N/A	N/A	N/A

a User-generated suicide content was collected along with source-specific metadata and previous content as context.

b N/A: not applicable.

c Q&A: question and answer.

##### Twitter

Twitter constitutes the majority of social media posts flagged for containing suicide-inducing information, with a substantial share of 74.69% [29]. To collect data related to suicide from Twitter, we used the Twitter application programming interface (API) v2 to gather posts that include suicide-related keywords in their text or hashtags. These suicide-related keywords were collected from previous research [31] and the guidelines of the “Korean Suicide Inducing Information Monitoring Group” [32]. We gathered 12,021 tweets, including 3635 with images, from May to August 2023 using the Twitter API. The suicide-related keywords used in the Twitter API are summarized in Table 3.

**Table 3.** Suicide-related keywords^a^.

Classification of search terms	Search terms	Synonyms	Korean version of search terms	Korean version of synonyms
Suicide-related terms	Suicide	Suicide	자살	자살
	Suicide method	Suicide method How to suicide How to commit suicide	자살방법	자살방법 자살하는법 자살하는방법
	Dying method	Dying method How to die	죽는방법	죽는방법 죽는법
	Suicidal idea	Suicidal idea Suicidal thinking	자살충동	자살충동 자살생각
	Fall down	Fall down Suicide by jumping from a height	투신	투신 투신자살
	Hanging	Hanging Hanging suicide Neck hanging	목맴	목맴 목맴자살 목매달기
	Will	Will ·How to write will	유서	유서 유서쓰는법
Self-harm–related terms	Self-harm	Self-harm	자해	자해
	Self-harm method	Self-harm method How to self-harm	자해방법	자해방법 자해하는법
	Wrist cutting	Wrist cutting How to cut my wrist Wrist cutting method	손목자해	손목자해 손목자해하는법 손목자해방법
	Self-harm wound	Self-harm wound Self-harm mark Treatment for self-harm wound	자해흉터	자해흉터 자해자국 자해흉터치료
	Drug overdose	Drug overdose Drug lethal dose	약물과다복용	약물과다복용 약물치사량
	Acetaminophen	Acetaminophen overdose Acetaminophen lethal dose	타이레놀	타이레놀과다복용 타이레놀치사량
Suicide risk factor terms	Academic score	Academic concern Academic score	성적	성적 성적고민
	Bullying	Bullying Covert bullying Outcast	왕따	왕따 은따 따돌림
	School violence	School violence	학교폭력	학교폭력 학폭
	Family troubles	Family troubles	가족문제	가족문제
	Domestic violence	Domestic violence	가정폭력	가정폭력
	Dropout	Dropout How to drop out Dropout method	자퇴	자퇴 자퇴하는법 자퇴방법
	Career	Career Career concern	진로	진로 진로고민
Suicide prevention terms	Suicide prevention	Suicide prevention	자살예방	·자살예방
	Call for life	Call for life Call for life of Korea	생명의전화	생명의전화 한국생명의전화
	Suicide prevention center	Suicide prevention center 1393	자살예방센터	자살예방센터 1393
	Psychiatry	·Psychiatry ·Neuropsychiatry Psychiatry department Mental hospital	정신과	·정신과 ·신경정신과 ·정신건강의학과 정신병원
	Mental health center	Mental health center	정신건강복지센터	정신건강복지센터
Depression-related terms	Depression	·Depression ·Depressed ·Depressive disorder ·Depressive symptom	우울증	우울증 우울 ·우울장애 ·우울증상

a Search terms and synonyms are defined in the study by Park et al [33].

##### Q&A Platform

On Q&A platforms, users often post questions about suicide-related issues, such as suicide methods, or respond to these queries. We collected questions and answers containing suicide-related keywords from Naver Knowledge In (a Korean Q&A platform) [33]. We collected data from March 2022 to March 2023, using the same keywords as those used for Twitter, resulting in 13,104 content items.

##### Online Support Forum

In online support forums, people write about their suicide-related concerns, and counselors provide responses to support them [31]. We collected posts from Lifeline Korea [34] and the Companions of Life Suicide Prevention Counselling [35]. We collected 17,325 pieces of content posted from March 2021 to June 2023.

##### Online Community

DCinside [36], a widely used online community in Korea comparable to Reddit, includes boards that function similarly to subreddits. We collected posts from 2 depression-focused boards (depression-minor and depression-mini boards) on the DCinside, known to contain suicide-related posts and where actual suicide incidents have been reported [37]. We collected posts including those containing images, resulting in a total of 794 data entries. Overall, we collected 43,244 entries of harmful suicide content, in total, from 4 online sources, with each harmful suicide content entry comprising content, previous content, and metadata.

### Data Preprocessing

To remove private information from the benchmark and generate information that requires human intervention, such as link or image descriptions, we took the following steps. First, we removed all PII. This involves replacing URLs, names, locations, phone numbers, emails, and IDs within the text with corresponding tags. Thereafter, we provided supplementary descriptions for the contents of the external links. Given that these links may contain significant information for accurately understanding the content, we manually reviewed the links and summarized their content. Third, we added text descriptions to the images whenever they were included in the content. We used GPT-4 to generate initial descriptions, which were subsequently reviewed and refined for accuracy by the researchers. Consequently, all PII values were removed from the text of the data, and we created link descriptions that summarized the content of any URLs present in the content text, along with text descriptions for the images.

### Data Annotation

#### Task Description Document

The task description document was designed to explain the harmful suicide content detection and to provide guidance to the annotators. It contains vital information, including the purpose of identifying harmful suicide content and a detailed guide for annotating the content. In addition, it outlines the categories and subcategories of harmful suicide content, supplemented with real-world examples.

Our basis for understanding the definitions, categories, and examples of harmful suicide content was the “Korean Suicide Prevention Law” [2] and documents published by the “Korea Life Respect Hope Foundation’s suicide/harmful information monitoring team” [32]. We found that certain category names and descriptions were unclear or overlapped, thus requiring more distinct clarifications. To address this, we involved medical professionals in the data annotation process, which led to significant revisions and refinements of the categories and their descriptions, as well as the expansion of examples for each category. Following the studies by Fiesler et al [38] and Moon et al [39], we used an iterative coding process such that the medical experts individually annotate the real-world content, come together to refine the task description document, and then repeat the coding process individually. This updating process was iterative and performed 3 times to ensure comprehensive refinement. Further details of the iterative process are presented in the “Annotation Process” section. We demonstrate each category and its description in Table 4.

**Table 4.** Name and description of each suicide category.

Name	Description
Illegal suicide content	Content that can actively encourage others to commit suicide or assist suicide behavior.
Harmful suicide content	Harmful content that is not as harmful as illegal suicide content but clearly has the effect of causing suicide or self-harm in the general public.
Potentially harmful suicide content	Content that may trigger suicide or self-harm in some people but may not cause it in others or may rather have a positive effect in others.
Harmless suicide content	Content that is not harmful, such as content that helps prevent suicide to the general public or provides neutral information related to suicide.
Nonsuicide content	Content unrelated to suicide.

#### Annotation Process

The annotation process was divided into 3 phases. In each phase, medical experts (a clinical expert with an MD degree and a psychiatry professor with a PhD degree) annotated real-world harmful suicide content, using the task description document as a reference. At the end of each phase, the authors and annotators reviewed and enhanced the task description document through discussions, before proceeding to the next phase.

**Figure 3.**
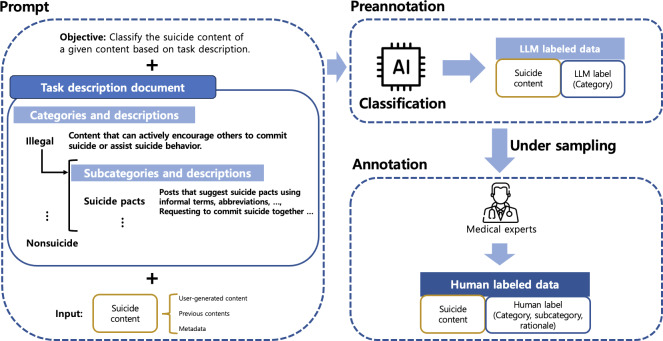
Illustration of the preannotation process. We construct prompts from the task description document, which includes categories, detailed descriptions, and subcategories (Table 4), and we use LLMs such as GPT-3.5 and GPT-4 to generate LLM-labeled data. We then sample the data to balance category distributions and have medical experts annotate them to build the human-labeled benchmark. AI: artificial intelligence; LLM: large language model.

In the first phase, medical professionals annotated harmful suicide content by referring to the initial task description document. Before starting the annotation, we preannotated and sampled the contents to be annotated from the data collections, as illustrated in Figure 3. Although the contents are gathered using suicide-related keywords, only a small fraction is actually harmful suicide content that can cause harm to others. Therefore, we used the task description document as instruction for the LLMs, allowing them to preliminarily categorize the content into predefined categories. This approach enhances the efficiency of the annotation process for medical experts and reduces mental strain and costs. Consequently, we used the OpenAI GPT API to preannotation and sampled 196 harmful suicide content for human annotation from the collected 2272 Twitter data, 17,325 online forum data, and 13,104 Q&A data. Medical professionals then proceeded to annotate the sampled 196 harmful suicide content by following the annotation protocol and the initial task description document. The annotation protocol is described in the later part of this section. During the annotation process, they did not refer to the LLM labels generated by the LLM. Following the annotation, both the categories and subcategories were updated, leading to a revision of the task description document. Specifically, we refined 7 subcategories, added 2 new ones, and removed one.

In the second phase, we diversified the harmful suicide content in the benchmark and refined the task description document. Before annotation, we further preannotated and sampled 175 harmful suicide content for annotation from a pool of 8408 Twitter data points collected between May and June 2023. Similar to the first phase, we preannotated them using OpenAI GPT API with instructions written based on the task description document. Subsequently, medical professionals began the annotation of sampled harmful suicide content, strictly adhering to the annotation protocol and using the revised version of the task description document as their guide. Once the annotation process was completed, we merged the 4 subcategories into 2.

In the final phase, we added multimodal (text and image) harmful suicide content to the benchmark dataset and included online communities as an additional source domain. For the image content, we initially generated textual descriptions of harmful images using visual language LLMs (“Data Preprocessing” subsection). These initial descriptions were then revised to correct any inaccuracies or fill in missing details. The refined descriptions were subsequently used to preannotate the content into categories and subcategories, as defined in the task description document from the second phase. Following this process, we preannotated and sampled 95 multimodal harmful suicide content items for annotation. Medical professionals then annotated based on the annotation protocol, and the task description document was finalized by revising the previous version.

Finally, we manually verified the entire benchmark dataset. This involved identifying and eliminating any remaining PIIs from all harmful suicide content and validating the final labels. During the finalization process, 14 content items were excluded from the benchmark. These contents deal with subcultures (such as games and comics) and, therefore, are incomprehensible to all annotators and cannot be categorized into any suicide category, leading to their exclusion. In addition, the task description document was completed, providing comprehensive information on the 5 categories and 25 subcategories, including their harmful category names, descriptions, and illustrative examples.

#### Annotation Protocol

In every phase, we adopted a consensus-based method for biomedical research and clinical practice [40,41]. For each harmful suicide content, 2 separate medical professionals (a clinical expert with an MD degree and a psychiatry professor with a PhD degree) independently labeled the category, subcategory, and rationale for their decisions regarding both the category and the subcategory. Each individual annotator assigned the label based on a comprehensive review of the user-generated content (text and image), previous content, and metadata associated with the harmful suicide content. The Inter-Annotator Agreement for category labels reached a high agreement of 0.77 (Cohen κ) after the second phase of the annotation process. In cases where there is a discrepancy in the category label assigned by individual annotators, a consensus is established through the input of 3 annotators, which includes an additional clinical expert (a psychiatry professor with a PhD degree). During this consensus, rationales written by the 2 individual annotators are combined into a single rationale. In addition, annotators comment on any data whose association with suicide content is uncertain, as well as on instances that imply a potential need to revise the task description. These comments were used at the end of each annotation phase to refine and update the task description document.

### Construction of English Benchmark

We further created an English benchmark by translating all input attributes (content text, link or image descriptions, and metadata such as user descriptions). To enhance the usability of the Korean harmful suicide content detection benchmark, it was translated into English using machine translation with GPT-4‐0613 for each attribute. As a result, we produced an English harmful suicide content detection benchmark containing the same 452 data entries as the original Korean harmful suicide content detection benchmark. The quality of the English benchmark is evaluated in detail in the “English Benchmark Validation” subsection.

## Results

### Overview

After constructing the harmful suicide content detection benchmark through the data processing workflow, we evaluated the dataset from 3 perspectives. First, we assess whether the categories and descriptions of harmful suicide content outlined in the task description document are appropriate for providing sufficient information as instructions to LLMs to classify suicide contents (“Leveraging Task Description” subsection).

Next, we evaluate whether the data modalities included in the benchmark (eg, images) are suitable for classification by LLMs (“Leveraging Multimodality” subsection). In addition, we verify whether the LLMs can perform classification using only few-shot examples without further training through few-shot experiments (“Leveraging Few-shot Examples” subsection).

Finally, for the English harmful suicide content detection benchmark, we conduct experiments using various LLMs to compare performance with the original Korean benchmark to verify the use and carry out a direct analysis of data quality to verify the translation’s quality (“Comparison Between LLMs” subsection and “English Benchmark Validation” subsection).

#### Setup

We used the GPT-3.5-turbo-16k API with a temperature of 0.0 and default hyperparameters, conducting 3-8 runs to calculate the average and standard error. For the few-shot experiments, we adopted an *N*-way *K*-shot approach by selecting *K* samples from each of the 5 classes (n=5) in the training dataset (“Data Annotation” subsection).

#### Metrics

We used the following metrics:

1. Macro *F*_1_ measures the overall performance across the 5 categories.
2. *Mean Absolute Error (MAE) of Harmfulness* measures the model’s deviation in predicting harmfulness and is categorized into four levels: 3 (most harmful: Illegal Suicide Content), 2 (harmful: Harmful Suicide Content), 1 (potentially harmful: Potentially Harmful Suicide Content), and 0 (not harmful: Harmless Suicide Content and Nonsuicide Content). This metric assesses the extent of the error in terms of harmfulness.
3. *Illegal* identifies illegal suicide content, and it is crucial for prompt regulation.
4. Harmful separates illegal or harmful suicide content from noncritical content, which is essential for moderating the content that poses harm.

In alignment with our objective of identifying and moderating as much harmful content as possible, our model is designed to initially detect harmful content, after which the results are carefully reviewed by a human moderator or expert. Hence, recall is prioritized over precision. This focus is particularly important for the Illegal and Harmful content categories, where missing instances of harmful suicide content pose a far greater risk than misclassifying content that is actually safe. Within our proposed framework, human moderators or clinical experts make the final judgment, ensuring that such misclassified cases can be corrected while minimizing the chance of harmful content going undetected. This design reflects how the system can function in real-world moderation scenarios, where recall is prioritized to maximize safety without disregarding precision.

### Leveraging Task Description

We investigated the formulation of a task description document with diverse and extensive information into instructions because instruction construction significantly influences LLM performance [42-44]. The task description document for the harmful suicide content detection task contains crucial details, including the names and descriptions of 5 suicide categories as well as the names and explanations of 25 subcategories constituting up to 60% of the instruction at maximum. Category information includes detailed names and descriptions of the categories and subcategories. Thus, our experiments were designed to determine the details that most significantly impact performance by varying the granularity of the information.

#### Setup

We evaluate harmful suicide content detection performance by varying the detailed category information levels as follows: (1) category name; (2) category name and description; (3) category name with category description and subcategory name; and (4) category name with category description and subcategory name with subcategory description.

- Category name and description
- Category name with category description, and subcategory name
- Category name with category description, subcategory name with subcategory description

#### Results

Table 5 shows that the performance improves with more category information, with the most comprehensive level yielding the highest *F*_1_-scores. Specifically, the macro *F*_1_-score increases by 89% (from 18.86 to 35.75), and the illegal *F*_1_ and harmful *F*_1_-scores increase by 200% (from 11.87 to 35.80) and 57% (from 37.63 to 59.10), respectively, as compared with when using only the category name.

The increasing trend in macro *F*_1_ and illegal or harmful *F*_1_-scores suggests that more detailed information enhances the model’s detection capabilities. However, adding only category descriptions decreased illegal or harmful recall (from 27.27 and 65.77 to 20.00 and 57.66, respectively).

**Table 5.** Results from the category and subcategory information detail experiment^a^.

Category information	Subcategory information	Macro *F*_1_	MAE^b^	Illegal *F*_1_	Illegal recall	Harmful *F*_1_	Harmful recall
Name	N/A^c^	18.86 (0.08)	1.4809 (0.0044)	11.87 (0.04)	27.27 (0.00)	37.63 (0.11)	65.77 (0.00)
Name and description	N/A	25.13 (0.16)	1.2173 (0.0042)	13.23 (0.17)	20.00 (0.00)	39.18 (0.04)	57.66 (0.00)
Name and description	Name	32.18 (0.06)	0.9867 (0.0016)	26.36 (0.00)	30.91 (0.00)	45.26 (0.08)	66.67 (0.00)
Name and description	Name and description	35.75 (0.29*)^a^*	0.8549 (0.0079**)**^a^	35.80 (0.87**)**^a^	58.79 (1.21**)**^a^	59.10 (0.41**)**^a^	86.79 (0.60**)**^a^

a A consistent increase in macro *F*_1_, illegal *F*_1_, and harmful *F*_1_-scores is observed as the amount of information increases. Values are mean (SE). These values indicate the best performance in each column.

b MAE: mean absolute error.

c N/A: not applicable.

### Formulating LLM Inputs

We assessed performance changes by incorporating images and training examples as inputs. We focused on the impact of images as multimodal data (“Leveraging Multimodality” subsection) and the effect of using training data with the annotation guide as postinstruction when combined with instruction (“Leveraging Few-shot Examples” subsection).

### Leveraging Multimodality

#### Setup

The objective of this experiment was to determine the effect of image information on the classification performance of the model. We used 2 methods of conveying image information and compared their performances: the first method converts images into text descriptions, referred to as image description, whereas the second uses the images directly as inputs, referred to as vision. Three settings were tested for image descriptions: the first did not provide any image information, the second generated image descriptions using a model (gpt-4‐1106), and the third involved human modifications to the descriptions created by the model. This allowed for a comparison of the performance of the models based on the generation of text-based image descriptions. In addition, we examined the impact of images (vision) when paired with the same image descriptions to observe their influence on performance. This involved adding the original image to each image description experiment for comparison. Overall, this setup evaluates the model’s performance in terms of the modality of suicide content through image descriptions and assesses the model’s multimodal capabilities through vision. Notably, during the annotation process, the annotators labeled the suicide category of the content based on both the text and the original images. We used GPT-4-turbo-2024-04-09, which can use both text and image inputs, for this experiment. We conducted an experiment on 113 test data entries that included images, among which only 3 belonged to the illegal suicide category; thus, illegal metrics were excluded from the results.

#### Results

Table 6 shows the impact of multimodal information on harmful suicide content detection tasks. In experiments regarding image descriptions without visual information, providing image details leads to superior performance compared with omitting them. Specifically, when using GPT-4–generated image descriptions, macro *F*_1_ increased by 9.16% (from 50.46 to 55.08) and MAE decreased by 15.93% (from 0.3894 to 0.3333), indicating enhanced classification performance across all suicide categories. In addition, harmful *F*_1_ and recall both increased by 8.00% (from 68.50 to 73.98 and from 75.76 to 81.82), suggesting that image information significantly aids in identifying harmfulness within suicide content. Comparing GPT-4 and human-modified image descriptions, using human-modified descriptions results in a reduction of macro *F*_1_ by 3.55% (from 55.08 to 53.19) and increases MAE by 19.4% (from 0.3333 to 0.3982), although harmful *F*_1_ increases by 2.61% (from 73.98 to 75.91) and harmful recall by 16.66% (from 81.82 to 95.45), indicating that human modifications enhance clarity and detection of harmfulness in content while decreasing overall category performance.

**Table 6.** Results of the input modality experiment on a subset of the benchmark that includes images^a^.

Image description (text)	Vision (image)	Macro *F*_1_	MAE^b^	Illegal *F*_1_	Illegal recall	Harmful	Harmful recall
No description	X	50.46 (0.12)	0.3864 (0.0135)	N/A^c^	N/A	68.50 (0.21)	75.76 (0.26)
GPT-4 description	X	55.08 (0.09)^a^	0.3333 (0.0102)^a^	N/A	N/A	73.98 (0.09)	81.82 (0.00)
Human description	X	53.19 (0.13)	0.3982 (0.0154)	N/A	N/A	75.91 (0.08)^a^	95.45 (0.00)^a^
No description	O	47.78 (0.05)	0.4189 (0.0102)	N/A	N/A	67.42 (0.07)	90.91 (0.00)
GPT-4 description	O	50.08 (0.16)	0.3894 (0.0234)	N/A	N/A	69.22 (0.34)	83.33 (0.26)
Human description	O	46.55 (0.15)	0.4631 (0.0270)	N/A	N/A	69.37 (0.01)	90.91 (0.00)

a We compare 3 types of image descriptions (none, GPT-4-generated, and human-modified) with and without vision input. Without vision, GPT-4 descriptions achieve the best macro *F*_1_ and MAE, while human descriptions perform best on harmful metrics. With vision, we observe an overall decrease in performance. Values are mean (SE). These values indicate the best performance in each column. “O” indicates that the column is used (vision) and “X” indicates that the column is not used.

b MAE: mean absolute error.

c N/A: not applicable.

In experiments using image information as visual input, we found a general decrease in overall performance across all settings, with reductions in macro *F*_1_, MAE, and harmful *F*_1_. Even in scenarios without image descriptions, macro *F*_1_ decreased by 5.31% (from 50.46 to 47.78), and MAE increased by 8.41% (from 0.3864 to 0.4189). Particularly, the *F*_1_-scores of potentially harmful content decreased significantly (from 54.46 to 42.77). This is owing to the model’s sensitive reaction to certain images of potentially harmful suicide content, overestimating their harmfulness and classifying them as harmful.

However, in settings where we used images only as vision (no image description), the harmful recall score was 90.91, which was higher than when no image information was used (75.76); the score was 83.33 when using images with GPT-4 image description, which was higher than when using GPT-4 image description alone (81.82). This suggests that despite a decrease in the overall model performance owing to multimodality, using image information improves the identification of harmfulness in suicide content. In addition, vision can convey more information about harmfulness than text descriptions when human modification does not explicitly note the harmfulness of an image. Overall, the experiments with image descriptions confirmed that the information contained in an image enhances model performance in the harmful suicide content detection task, whereas adding vision information in a multimodal format decreases performance. However, the increase in harmful recall when using vision supports the potential of using vision as an effective tool for enhancing model capabilities in identifying harmful content, paving the way for future improvements in multimodal model performance.

### Leveraging Few-Shot Examples

Few-shot refers to a setting where the model is provided with a small number of labeled examples as demonstrations before classifying new instances [45]. The goal of our few-shot experiments is to assess whether LLMs can effectively learn from only a handful of examples in high-stakes domains, where large-scale annotation is often insufficient to train models.

#### Setup

We examined 1- to 5-shot configurations, corresponding to 1-5 examples per category, totaling 5-25 examples. To construct the pool of demonstrations, medical experts who participated in the benchmark annotation selected 10 representative samples per category (50 in total). These expert-selected samples were used as the source for training demonstrations, while the remaining annotated data served as the test set. From the pool, 5 examples per category were randomly drawn for each experiment. To ensure robustness, we repeated the same experiment with 3 different random seeds, each sampling distinct few-shot samples, and we report the averaged results across these runs.

#### Results

**Figure 4.**
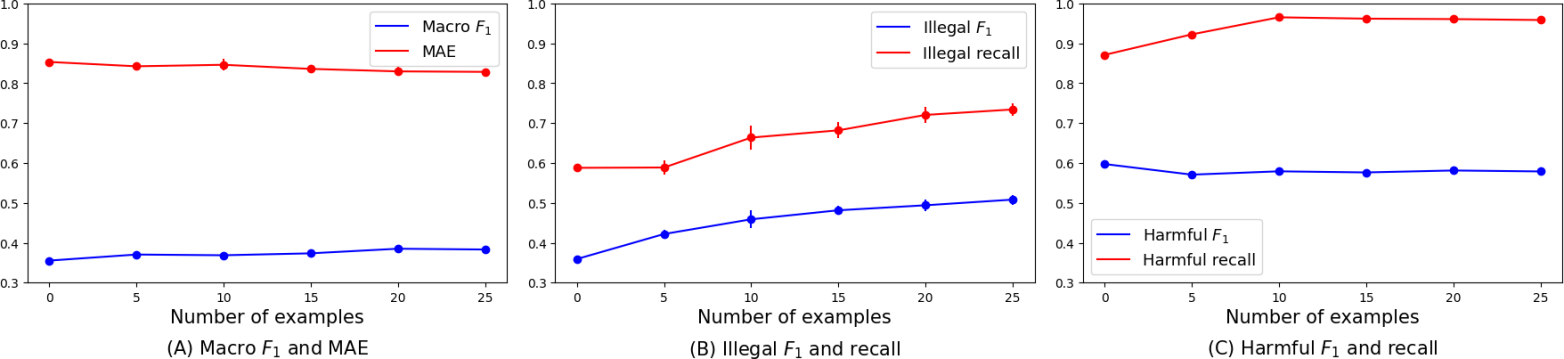
Results from the few-shot example experiment. Increasing examples increases illegal *F*_1_ and recall, with 5-shot setting achieving peak performance in the illegal metric. (A) Macro *F*_1 _and mean absolute error; (B) illegal *F*_1_ and recall; and (C) Harmful *F*_1_ and recall. MAE: mean absolute error.

Figure 4 shows how the performance metrics changed with the number of demonstration examples, with the standard error represented by vertical bars for each few-shot case. As the number of examples increased, macro *F*_1_, MAE, and illegal metrics improved (Figure 4A), specifically illegal *F*_1_ and recall in the 5-shot (Figure 4B). Although the *F*_1_-score for harmful effects remained relatively stable, recall increased but plateaued after a certain threshold (2-shot) (Figure 4C).

### Comparison Between LLMs

We compared the performance of various LLMs in identifying harmful suicide content. Because open-sourced LLMs have instruction-following capabilities that depend on the language they have seen in the instruction tuning phase, we conducted experiments with different models for Korean and English benchmarks to address language barriers.

#### Setup

We categorized the selected LLMs into closed and open-sourced models. For the Korean benchmark, we used closed models because of the lack of open-source or multilingual LLMs that can properly follow the task’s instructions in Korean. We also included a random baseline that arbitrarily categorized content into one of the 5 categories.

### Closed Models

We used OpenAI’s GPT-3.5 (gpt-3.5-turbo-16k-0613) and GPT-4 (gpt-4‐1106-preview), which are accessed through the OpenAI API and capable of handling a context length of 1,28,000 characters. In addition, we experimented with Clova X, an LLM trained on Korean, using the Naver API [46].

### Open-Sourced Models

We used the Zephyr-7B-beta model [47], an enhanced version of mistral-7B, which supports a context length of up to 32,000 characters. We also use LongChat-7B-16k [48] and Vicuna-7B-v1.5‐16k [49], which are both fine-tuned Large Language Model Meta artificial intelligence (AI) models with a maximum context length of 16,000 characters.

To explore the model’s adaptability of the model in few-shot learning contexts, we conducted experiments in both the zero-shot and 5-shot scenarios (“Leveraging Few-Shot Examples” subsection). However, for models unable to accept the context length of 12k tokens required for the 5-shot experiments, such as Clova X (4096), we limited our analysis to the zero-shot trials.

#### Results

**Figure 5.**
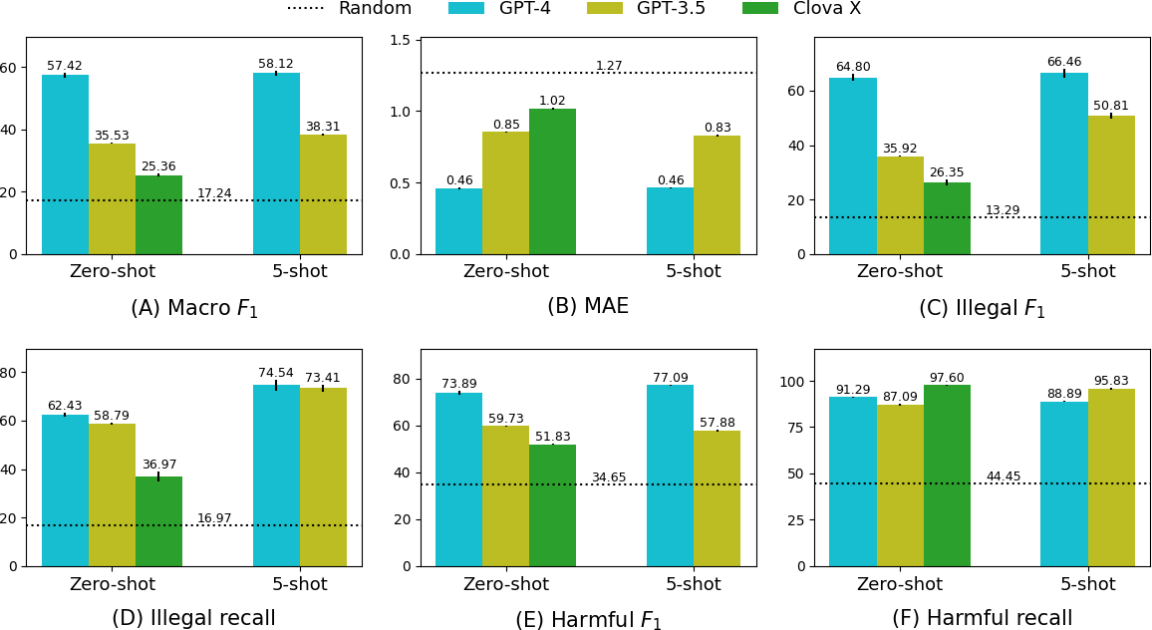
Results from the Korean benchmark experiment. Hatched bars indicate the Korean large language model (Clova X). Although Clova X has lower overall performance than generative pretrained transformers, it excels in harmful recall. (A) Macro *F*_1_; (B) mean absolute error; (C) illegal *F*_1_; (D) illegal recall; (E) harmful *F*_1_; and (F) harmful recall. MAE: mean absolute error.

Figure 5 shows the performance of the GPT models and Clova X on the Korean benchmark. GPT-4 outperformed all other models in every metric except for harmful recall (Figure 5A-E). GPT-3.5 follows GPT-4 in terms of performance across all metrics, except for harmful recall. Clova X showed lower performance than the GPT models but achieved the highest score in harmful recall (Figure 5F), indicating its high sensitivity to harmful content.

**Figure 6.**
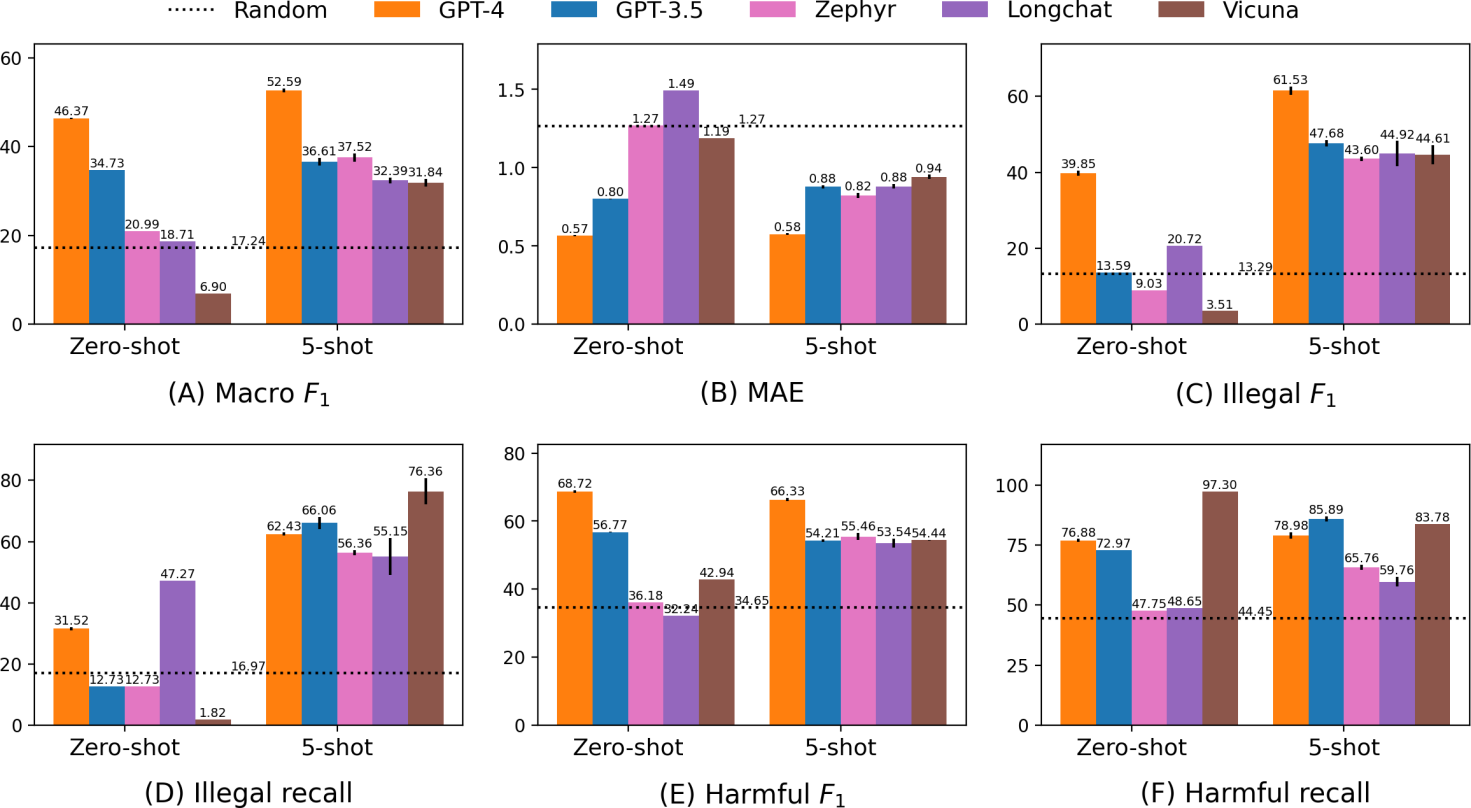
Results from the translated English benchmark experiment. Closed models (GPT-4 and GPT-3.5) show superior performance in the zero-shot setting compared with open-sourced models, whereas open-sourced models reach comparable performance to GPT-3.5 in 5-shot. (A) Macro *F*_1_; (B) mean absolute error; (C) illegal *F*_1_; (D) illegal recall; (E) harmful *F*_1_; and (F) harmful recall. MAE: mean absolute error.

Figure 6 shows the performance of the GPTs and open-sourced LLMs on the translated English benchmark. GPT-4 exhibited the highest performance in differentiating categories in both the zero-shot and 5-shot settings across various metrics (Figure 6A-C and E). It leads the performance charts with macro *F*_1_-scores of 46.37 in zero-shot and 52.59 in 5-shot. Notably, GPT-4 showed a significant MAE difference (0.5655 in zero-shot and 0.5755 in 5-shot), indicating that even when the category predictions were incorrect, they tended to be within similar levels of harmfulness.

GPT-3.5 ranked second to GPT-4 in category distinction performance (Figure 6A, B, and E) in zero-shot settings and showed comparable performance to open-sourced models in 5-shot settings. Its recall was relatively higher than GPT-4 in 5-shot settings (Figure 6C-F), indicating a more sensitive response to harmful information than the GPT-4.

Zephyr outperforms random in zero-shot settings with a macro *F*_1_ of 20.99 and MAE of 1.2711; in addition, it achieves a comparable performance to GPT-3.5 in 5-shot settings with macro *F*_1_ of 37.52 and MAE of 0.8217 (Figure 6A and B ). LongChat exhibits the largest standard error in the illegal and harmful metrics (Figure 6C-F), indicating that few-shot examples significantly impact performance compared with other models. It recorded the highest standard errors in illegal *F*_1_ and harmful *F*_1_ at 4.09 and 1.67, respectively (Figure 6C and E). LongChat also showed the lowest recall for illegal and harmful content, particularly in harmful content, suggesting that it is less sensitive to harmful information (Figure 6D and F). Vicuna recorded the lowest performance in category classification (Figure 6A) among all models but achieved high recall for illegal and harmful content (Figure 6D and F). Notably, it scored the highest illegal recall of 76.36 and a harmful recall of 83.78, comparable with GPT-3.5’s 85.89.

### Analysis

#### Open-Sourced Versus Closed LLMs

GPT-4 recorded the highest performance across all accuracy metrics (macro *F*_1_, MAE, illegal *F*_1_, and harmful *F*_1_) for all few-shot settings. In 5-shot settings, open-sourced models achieve a similar performance to GPT-3.5. However, in zero-shot settings, they struggled to understand lengthy instructions, resulting in random predictions (eg, LongChat) or biased predictions toward specific categories (eg, Vicuna), with Zephyr slightly outperforming random. In 5-shot scenarios, Zephyr matches GPT-3.5 in macro *F*_1_, MAE, and harmful *F*_1_, whereas LongChat and Vicuna show comparable performance in their respective metrics. Except for Vicuna, open-sourced models generally showed lower recall than closed models in terms of illegal and harmful content.

### Original Korean Versus Translated English

**Figure 7.**
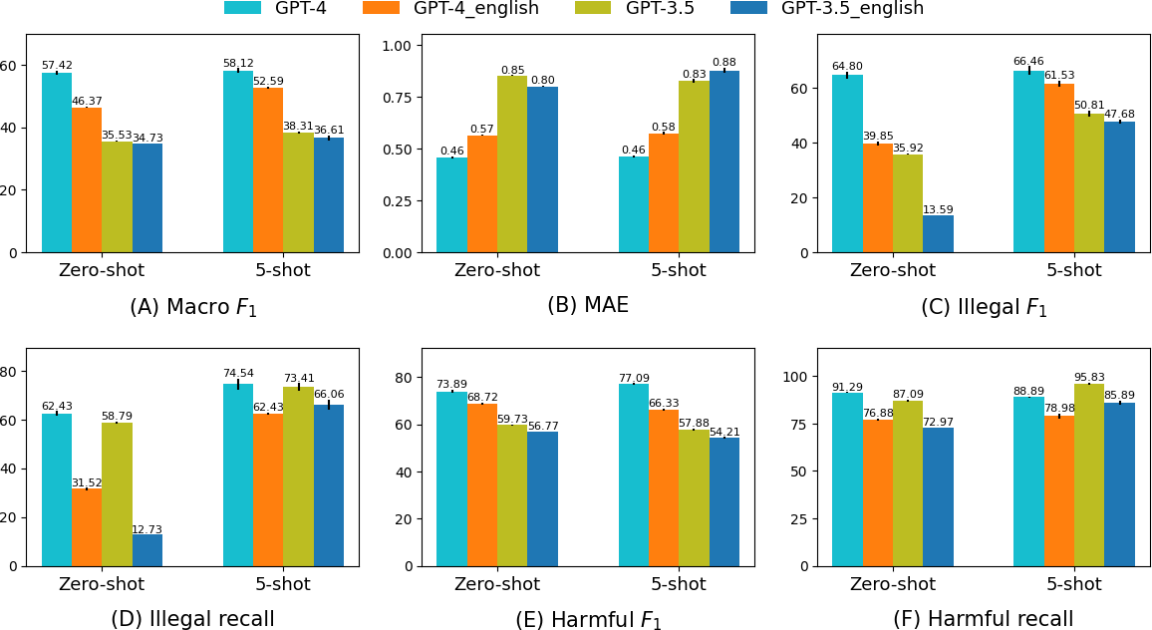
Performance comparison of closed models (GPT-3.5 and GPT-4) on the Korean and translated English benchmarks. Closed models exhibit better classification performance on the Korean benchmark than on the English benchmark, with the most significant difference noted in classifying the illegal category. (A) Macro *F*_1_; (B) mean absolute error; (C) illegal *F*_1_; (D) illegal recall; (E) harmful *F*_1_; and (F) harmful recall. MAE: mean absolute error.

Figure 7 shows an analysis of GPT-3.5 and GPT-4’s performance on the Korean and translated English benchmarks. Both models performed better on the Korean benchmark across all *F*_1_ metrics (Figure 7A, C, and E). However, GPT-4 shows a decrease in macro *F*_1_ from the English to the Korean benchmark by 19.24% in zero-shot (from 57.42 to 46.37) and 9.51% in 5-shot (from 58.12 to 52.59), with the largest decrease in illegal *F*_1_ by 36.17% in zero-shot (from 64.80 to 39.85). GPT-3.5 also showed a considerable reduction in zero-shot illegal *F*_1_ by 62.17% (from 35.92 to 13.59). Illegal recall decreases considerably, with GPT-4 decreasing by 49.51% (from 62.43 to 31.52) and GPT-3.5 by 78.34% in illegal recall (from 58.79 to 12.73), indicating a larger decrease than in *F*_1_-scores (Figure 7D). The decrease in harmful *F*_1_ is less significant, with GPT-4 decreasing by 7.00% (from 73.89 to 68.72) in zero-shot, and GPT-3.5 decreasing by 4.96% (from 59.73 to 56.77). This indicates that while translating the benchmark using GPT-4 does not significantly affect the overall quality, it may lead to issues in specific categories, notably illegal.

### English Benchmark Validation

Considering the use of the English benchmark for experimenting with open-sourced models, it is necessary to evaluate the translation results. In particular, for content containing words related to suicide and harmful information, it is crucial to assess both the overall translation quality and how well the content has been translated. Because OpenAI’s use policy potentially refuses to respond to harmful content, there may be instances in which proper translation has not been achieved. Therefore, we analyzed the following 2 aspects: (1) overall translation quality (quantitative analysis), and (2) translation of harmful content (qualitative analysis).

We evaluated the text content (CONTENT_TEXT) of every instance in the benchmark because every instance contains content text and it constitutes the largest proportion of text. We performed a quantitative analysis to assess translation quality using models (GPT-4‐0613) and a qualitative analysis of the translation of harmful content by the authors.

*Translation quality (quantitative analysis)*: Translation quality assesses how similar the translated content (in English) is to the original content (in Korean). We evaluated translation quality using Scalar Quality Metric (SQM) and Direct Assessment (DA) methods [50] through the GPT-4‐0613 API, which aligns closely with human evaluations. SQM evaluates the translation quality of the source text (Korean) and target text (English) on a scale of 0-100, with descriptions provided for “no meaning preserved,” “some meaning preserved,” “most meaning preserved and few grammar mistakes,” and “perfect meaning and grammar.” DA, such as SQM, rates translation quality on a scale of 0-100 but provides descriptions only for “no meaning preserved” and “perfect meaning and grammar.” On average, the translated contents scored 79.55 on SQM and 78.10 on DA, indicating that most instances of the benchmark translation results fall under “most meaning preserved and few grammar mistakes,” successfully retaining the original meaning.

*Translation of harmful content (qualitative analysis)*: Illegal and harmful suicide content includes harmful words and expressions related to suicide and self-harm, encompassing abbreviations, drug names related to suicide, and expressions of methods for suicide and self-harm. Moreover, owing to OpenAI’s use policy, there are cases in which harmful content is not translated or translation is refused. Thus, to determine how well such content was translated, we analyzed translation error cases for expressions related to suicide: (1) expressions related to suicide (abbreviations and words), and (2) OpenAI moderation.

*Expressions related to suicide*: After analyzing 55 instances of illegal suicide content and 56 instances of harmful suicide content, we identified the following types of translation errors: (1) abbreviation translation error, (2) translation of substances used for suicide, and (3) translation of slang related to suicide and self-harm.

Table 7 shows examples of translation errors for each category and error type. *Abbreviation translation error* occurs when abbreviations related to suicide and self-harm are incorrectly interpreted. To evade online platform moderation, abbreviations related to suicide are often used. In these cases, the translation process incorrectly translates these abbreviations into entirely different words. The Korean abbreviation means “commit double suicide and death leap”; however, the English translation misinterprets it entirely. In this study, 12 benchmark instances were identified. *Translation of Substances used for suicide* refers to cases in which drugs related to suicide and self-harm were incorrectly translated. Substances used for suicide are often referred to by abbreviations to avoid online platform moderation, and the actual drug names are often translated into general names for drugs during translation. In this example, the drug “zolpidem” was translated as “SleepingPill,” which translates to the purpose of the drug (zolpidem is a type of sleeping pill) rather than the actual name of the drug. However, such translations result in the inability of the model to correctly identify the sale of specific drugs (illegal suicide category) during the category classification process. In this study, 15 instances were identified. *Translation of slang related to suicide and self-harm *refers to errors in the translation of clear expressions of suicide. For example, the Korean expression for “bloodletting self-harm” was incorrectly translated as “blood donation” in English, which changed the meaning of the text. Three instances were identified for this case.

**Table 7.** Qualitative analysis of benchmark translation results. The source text is the content text from the Korean benchmark data, and the proper translation is the result translated by a human while preserving the meaning. The translation result is obtained using a model and has been applied to the English benchmark. The underlined words indicate parts where translation errors occurred in the model’s output.

Type	Content text
Translation error in the abbreviation (illegal suicide content)	
Source text	ㄷㅂㅈㅅ ㅌㅅ 하실 분 자차나 면허 있으신 분으로요
Proper translation (human)	Looking for someone who will commit double suicide and death leap, preferably someone with a car or a driver’s license.
Translation result (model)	Looking for someone who can do delivery and sales, preferably someone with a car or a driver’s license.
Translation error in substances that are used for suicide (illegal suicide content)	
Source text	정품#졸ㅍㅣ뎀판ㅁㅐ 하는곳 가짜없고 사기없는 솔직한 비밀상담 관심空 #여성최음ㅈㅔ ㄱㅜ입하는 곳. 라인: [ID] 카톡: [ID] 텔레: [ID]
Proper translation (human)	#GenuinezolpidemSellingPlace A place where there are no fakes, no scams, honest confidential consultation. Interested in #FemaleAphrodisiac Purchase place. Line: [ID] Kakaotalk: [ID] Telegram: [ID]
Translation result (model)	#GenuineSpleepingPillSellingPlace A place where there are no fakes, no scams, honest confidential consultation. Interested in #FemaleAphrodisiac Purchase place. Line: [ID] Kakaotalk: [ID] Telegram: [ID]
Translation of slang related to suicide or self-harm (harmful suicide content)	
Source text	사혈자해를 해보고싶다는 생각이 들어
Proper translation (human)	I’m thinking about wanting to try bleeding self-harm.
Translation result (model)	I’m thinking about wanting to try blood donation.

### OpenAI Moderation

During the translation process of the benchmark data, we found a few instances where different translation errors occur from those related to expressions of suicide; some content are not translated at all. Unlike parts of the text that are translated incorrectly, the OpenAI API (gpt-4‐1106) refuses to translate and generates content (translated output) that has no relation to the original text (input text). Two instances occurred for this case; one involving content asking for help in committing suicide or self-harm and another involving direct sexual content.

## Discussion

### English Benchmark Integrity

The English benchmark was generated through machine translation using GPT-4. Quantitative evaluation showed relatively high scores (SQM: 79.55, DA: 78.10), indicating that most meanings were preserved. However, qualitative analysis revealed errors in suicide-related slang, abbreviations, and substance names (eg, bleeding self-harm translated as blood donation, zolpidem simplified to sleeping pill). Such inaccuracies may affect model performance and limit the benchmark’s validity for multilingual generalization. Therefore, results on the English benchmark should be interpreted with caution. Future work will involve human validation of high-risk categories and improved translation methods for suicide-specific terminology.

### Benchmark Size

Although the harmful suicide content benchmark is an essential step toward understanding and moderating online suicide-related content, it encompasses 452 data entries. This relatively small benchmark size is largely attributable to the fact that posts related to suicide comprise a small fraction of the total online content. In addition, filtering and deleting such content by online sources inherently limit the volume of data available for collection. Nevertheless, a carefully controlled annotation process that incorporates the knowledge of clinical experts supports the credibility of the benchmark and ensures a reliable set of labels. In addition, the task description document details 25 different subcategories of suicide content, and the benchmark comprises a wide array of suicide content, including actual data for each subcategory. Therefore, our detailed task description document and the data within our benchmark lay the groundwork for future efforts to create a large-scale suicide content dataset using the annotations described for the suicide content.

### Dataset Usage

The harmful suicide content benchmark includes a task description document containing categories and subcategories of suicide content, along with a dataset of 452 annotated entries. Users can leverage the contents of the task description document as instructions for LLMs. In addition, data labeled for training within the benchmark can be used as few-shot examples, while data labeled for testing can serve as evaluation data.

### Future Work

In clinical practice, detecting that a patient who has attempted suicide or self-harm has been exposed to suicide-related content can be highly valuable for preventing further self-harm or suicide attempts. This is because restricting harmful online content represents a relatively modifiable factor among the many risk factors associated with suicide and self-harm [51,52]. Building on this perspective, in future work, we plan to further validate the practical effectiveness of our suicide content detection model. Specifically, we aim to conduct a comparative study between individuals who have attempted suicide or self-harm and a control group of individuals without such experiences. By analyzing their online activity, we will investigate whether those with a history of suicidal or self-harming behaviors are more frequently exposed to harmful suicide-related content. Such a study would not only provide empirical evidence for the real-world use of our detection framework but also offer insights into the role of online harmful content in influencing vulnerable populations.

## Abbreviations

AI: artificial intelligence
API: application programming interface
DA: Direct Assessment
IRB: institutional review board
MAE: mean absolute error
PII: Personally Identifiable Information
Q&A: question and answer
SQM: Scalar Quality Metric
